# Editorial: Chronic inflammation and pharmacological interventions in cardiovascular diseases, volume II

**DOI:** 10.3389/fphar.2023.1322371

**Published:** 2023-11-13

**Authors:** Aiwei Yan, Xiaoping Wang, Can Cui, Min Zhang, Xianwei Wang

**Affiliations:** ^1^ Henan Key Laboratory of Medical Tissue Regeneration, Xinxiang Medical University, Xinxiang, China; ^2^ Department of Human Anatomy and Histoembryology, Xinxiang Medical University, Xinxiang, China; ^3^ School of Cardiovascular and Metabolic Medicine & Sciences, King’s College London BHF Centre of Research Excellence, London, United Kingdom

**Keywords:** chronic inflammation, cardiovascular diseases, pharmacological interventions, cell-based therapies, cellular and molecular mechanisms

## Introduction

Cardiovascular diseases encompass a variety of diseases affecting the heart and vascular system, including coronary artery disease, hypertension, and diverse cardiac disorders. These pathologies are frequently associated with numerous risk factors such as hypertension, high cholesterol, and diabetes (Prousi et al., 2023). Research has elucidated that these factors can often instigate chronic inflammation which can induce a series of adverse physiological reactions, thereby facilitating the occurrence and progression of cardiovascular diseases (Ferrucci and Fabbri, 2018; Goswami et al., 2021).

Presently, to counteract the detrimental effects of chronic inflammation on the cardiovascular system, researchers have initiated the exploration and implementation of various pharmacological intervention strategies (Li et al., 2023). Predominantly focusing on the utilization of anti-inflammatory drugs to attenuate the severity of chronic inflammation and its associated risks, these interventions aim to offer a more secure treatment regimen for patients with cardiovascular diseases (Delbaere et al., 2023).

**FIGURE 1:**
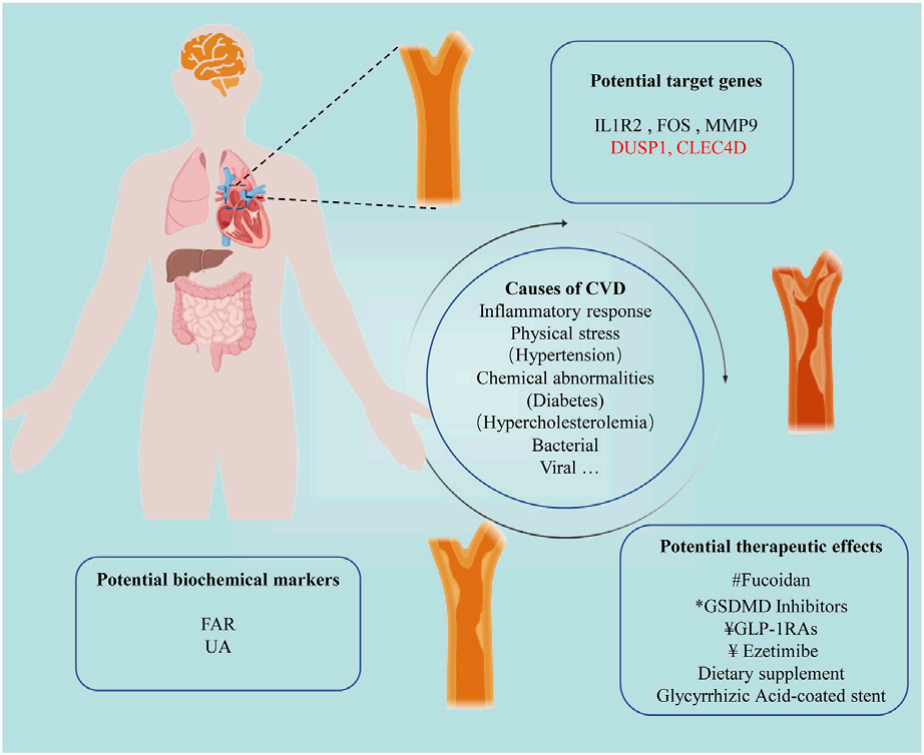
Illustrating New Discoveries in Cardiovascular Disease Treatment within this Topic Section. ^#^Natural product; *New Inhibitors; ^¥^Clinical medicine.

In the first series of studies centered on “*Chronic Inflammation and Pharmacological Interventions in Cardiovascular Diseases*”, a large number of studies were conducted. Given the sustained interest and critical importance of this area, we have initiated the second round of thematic discussions and eventually accepted eight original research papers and one review (Figure 1).

## Traditional techniques in cardiovascular disease treatment

Within this anthology of nine contributions, the mechanisms of clinical drug interventions in cardiovascular health continue to be unraveled with attention. To illustrate, Maganova et al. has delineated the beneficial effects of the dietary supplement fir terpenoids in enhancing cerebral blood flow and ameliorating arterial conditions indicative of biological aging. Concurrently, Wang et al. has cast a spotlight on the salutary properties of the natural entity FO in bolstering cardiac function. This is achieved through a marked reduction in Ang II-induced apoptosis, facilitated by the modulation of USP22/Sirt1 signaling pathways. Besides, Zhang et al. assessed the effect of GSDMD inhibitor Z-LLSD-FMK or Z-YVAD-FMK in diminishing vascular inflammation and hindering lesion progression in ApoE^−/−^ mice, a process orchestrated through the suppression of GSDMD activation. Drug-eluting stents (DES) have become a specific non-pharmacological therapeutic tool for the treatment of cardiovascular diseases (Crea, 2020). Augmenting the narrative on innovative medical devices, Teng et al. has presented a pioneering glycyrrhizin acid (GA)-coated stent, lauded for its inhibition of intimal hyperplasia and facilitation of re-endothelialization, thereby marking a significant breakthrough in cardiovascular therapy. Notably, when benchmarked against rapamycin-eluting stents, GA-eluting variants demonstrated a more extensive endothelial coverage, indicating a promising lead in therapeutic efficacy.

## Exploring cardiovascular therapies with big data

In the ongoing efforts to elucidate the intricacies of drug-disease interactions, big data analytics have emerged as an indispensable tool, underpinning a significant portion (5 out of 9) of the investigations encapsulated in this topic, leveraging this approach alongside detailed analyses of related disease reports to foster novel research avenues.

A notable investigation conducted by Chen et al. utilized a meticulous cross-sectional exploration of 413 individuals suffering from type 2 diabetes (T2D), uncovering a distinct positive correlation between the Fibrinogen albumin ratio (FAR) level in male patients and both brachial-ankle pulse wave velocity (baPWV) and arterial rigidity, thereby spotlighting potential avenues for further research in this area. Luo et al. embarked on a detailed analysis involving 225 individuals with coronary heart disease juxtaposed with a control group comprising 40 healthy individuals. Their focal point was discerning the interplay between serum uric acid (UA) levels and the severity as well as the treatment responsiveness in patients with PAH and congenital heart disease (PAH-CHD). The finding that serum UA could potentially function as a feasible and cost-effective biomarker for risk categorization and scrutinizing PAH-specific medicinal interventions stands as a testament to the depth of their investigation.

In parallel, Deng et al. employed network pharmacology to explore the underlying mechanisms through which GLP-1RAs reduce the incidence of myocardial infarction (MI) in T2DM patients. They underscored the multi-faceted role of GLP-1RAs in attenuating MI by modulating key biological targets and processes, and influencing cellular signaling pathways associated with atheromatous plaque development, myocardial remodeling, and thrombogenesis. Besides, Peng et al. undertook a holistic characterization of the peripheral whole blood transcriptome in individuals experiencing acute anaphylaxis and ST-segment elevation myocardial infarction (STEMI). This endeavor led to the identification of shared biological processes and immune cell landscapes, bringing to light critical hub genes, namely DUSP1 and CLEC4D.

Lastly, a meta-analytical systematic review and sequential trial scrutiny helmed by Chai et al. evaluated the influence of zetimide on the genesis of coronary atherosclerotic plaque composition. Their conclusions underscored zetimide’s potency in curtailing fibro-fatty plaque (FFP) formations, albeit without notable effects on fibrous plaque (FP), necrotic core (NC) or altering dense calcification (DC) dynamics.

These studies provide valuable insights and solutions to unsolved clinical questions. Instead of solely relying on basic laboratory research for validation, current investigations leverage big data analysis techniques, utilizing large-scale samples and a comprehensive perspective to address clinical issues, thereby ensuring the reliability and holistic understanding of the conclusions reached.

## Conclusion

Each investigation within this thematic compilation delineates critical pathways and prospective therapeutic strategies, shedding new light on advancements in the field of pharmacological interventions for cardiovascular diseases. These endeavors notably provide new insights for innovative treatments targeting chronic inflammation, thereby ushering in a new frontier in mitigating the adversities associated with these ailments.
